# Genome-wide RNAi screen for synthetic lethal interactions with the *C. elegans* kinesin-5 homolog BMK-1

**DOI:** 10.1038/sdata.2015.20

**Published:** 2015-05-12

**Authors:** André F. Maia, Marvin E. Tanenbaum, Matilde Galli, Daphne Lelieveld, David A. Egan, Reto Gassmann, Claudio E. Sunkel, Sander van den Heuvel, René H. Medema

**Affiliations:** 1 Instituto de Investigação e Inovação em Saúde, Universidade do Porto, Porto, Portugal; 2 Department of Medical Oncology, Laboratory of Experimental Oncology, University Medical Center Utrecht, 3584 CG Utrecht, The Netherlands; 3 Developmental Biology, Utrecht University, 3584 CH Utrecht, The Netherlands; 4 Department of Cell Biology, Cell Microscopy Center, University Medical Center Utrecht, 3584 CX Utrecht, The Netherlands

**Keywords:** Mitosis, RNAi, Caenorhabditis elegans, High-throughput screening

## Abstract

Kinesins are a superfamily of microtubule-based molecular motors that perform various transport needs and have essential roles in cell division. Among these, the kinesin-5 family has been shown to play a major role in the formation and maintenance of the bipolar mitotic spindle. Moreover, recent work suggests that kinesin-5 motors may have additional roles. In contrast to most model organisms, the sole kinesin-5 gene in *Caenorhabditis elegans, bmk-1*, is not required for successful mitosis and animals lacking *bmk-1* are viable and fertile. To gain insight into factors that may act redundantly with BMK-1 in spindle assembly and to identify possible additional cellular pathways involving BMK-1, we performed a synthetic lethal screen using the *bmk-1* deletion allele *ok391*. We successfully knocked down 82% of the *C. elegans* genome using RNAi and assayed viability in *bmk-1(ok391)* and wild type strains using an automated high-throughput approach based on fluorescence microscopy. The dataset includes a final list of 37 synthetic lethal interactions whose further study is likely to provide insight into kinesin-5 function.

## Background & Summary

The mitotic spindle consists of microtubule filaments that are organized into a bipolar configuration by a variety of microtubule-associated proteins (MAPs) and their regulators. Of the MAPs with motor activity, members of the kinesin-5 family play a central role. With four identical motor domains configured like a ‘dumbbell’^1^, kinesin-5 is capable of cross-linking and sliding apart antiparallel microtubules^2^, thereby generating an outward force on the spindle. In mammals, kinesin-5 is essential to separate centrosomes early in mitosis to form the bipolar spindle^3^, while in *S. cerevisiae* and *Drosophila* S2 cells kinesin-5 activity is used later in mitosis to elongate the anaphase spindle^4,5^. By contrast, the *C. elegans* kinesin-5 homolog BMK-1 acts as a break for spindle elongation in anaphase of the first embryonic division^6^. In this context, the cross-linking activity of BMK-1 likely resists the strong cortical forces in the one-cell embryo that pull the two spindle poles apart. Thus, while sequence conservation suggests that the basic activity of kinesin-5 is the same in all species, considerable variation exists in the requirement for this activity in mitosis. In addition, the dispensability of kinesin-5 for successful mitosis in *C. elegans* may hint at redundant mechanisms for spindle assembly in this organism.

Several lines of evidence suggest that kinesin-5 also has multiple roles beyond mitosis. Mammalian Eg5 is a regulator of axonal growth^7,8^ and has been shown to associate with ribosomes to enhance the efficiency of translation^9^, while *C. elegans* BMK-1 was identified in a genome-wide screen for genes that regulate germ cell apoptosis^10^.

**Figure 1:**
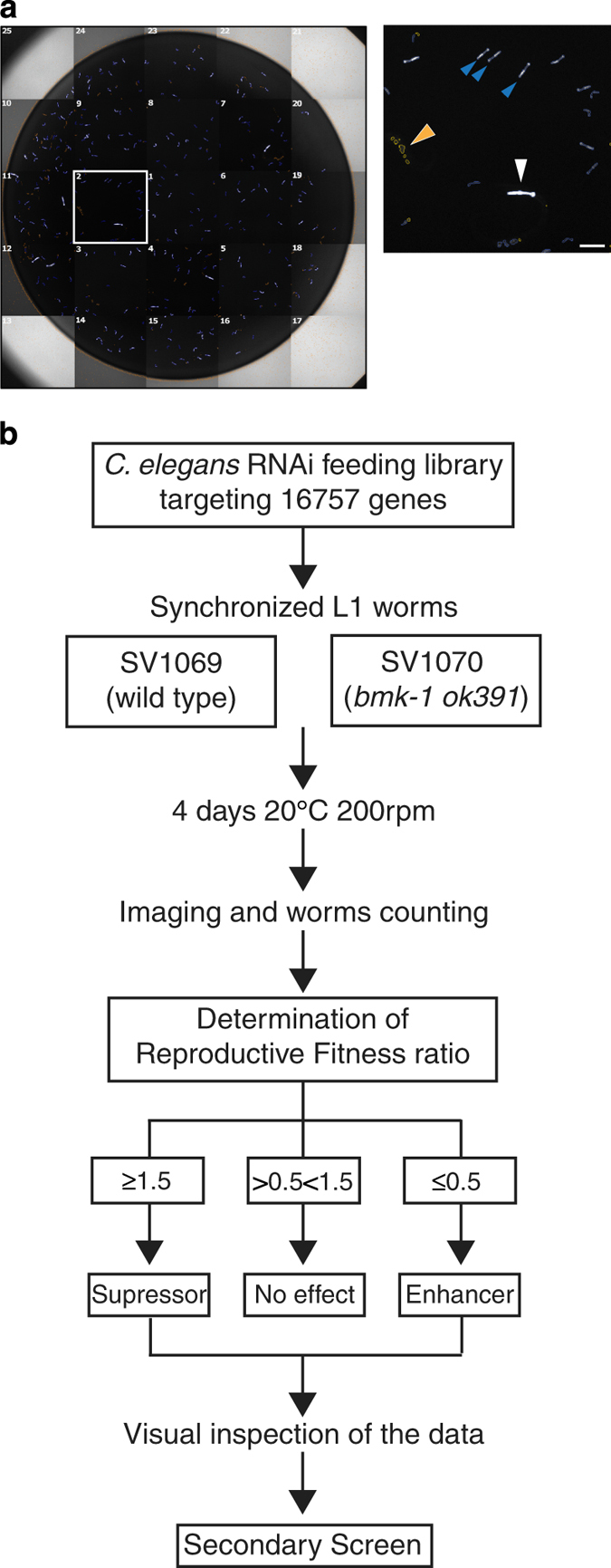
Experimental workflow. (**a**) Example of a mosaic reconstruction of an imaged well of the screen. In the magnification of field 2, the white arrowhead shows the pharynx of a parent worm, blue arrowheads show pharynxes of progeny, and the orange arrowhead points to excluded objects. Scale bar is 150 μm. (**b**) Flowchart of screening protocol. The primary screen was done in two technical replicates. The secondary screen followed the same protocol as the primary screen but used three biological replicates with two technical replicates each.

To gain insight into factors that may act redundantly with kinesin-5 in spindle assembly in *C. elegans* and to uncover possible additional functions of kinesin-5, we conducted a genome-wide synthetic lethal RNAi screen using animals harbouring the genetic deletion allele *bmk-1(ok391)*. The viability and fertility of *bmk-1(ok391)* animals is indistinguishable from wild type controls, making this genetic background ideal for a synthetic lethal screen. Knockdown by RNAi was carried out by bacterial feeding using the Ahringer genome-wide library^11^ adapted to a 96-well liquid format. We marked animals with green fluorescent protein in the pharynx (*myo-2::gfp*) for detection and accurate counting with an automated fluorescence microscopy system, in which adult worms (parents) and larvae (progeny) could be distinguished by the size of their pharynx. This allowed determination of ‘reproductive fitness’, defined as the number of progeny per parent. The primary screen consisted of two technical replicates per bacterial clone (16757 clones) expressing a gene-specific dsRNA. Genes that when knocked down significantly decreased reproductive fitness in *bmk-1(ok391)* animals relative to control animals were considered hits. These were selected for secondary screening (Fig. 1), which consisted of 3 biological replicates, each carried out in 2 technical replicates. Data analysis and validation steps generated a list of 37 genes that display synthetic lethality with *bmk-1(ok391)* (Data Records 1 and 2). The list contains genes implicated in both embryonic and post-embryonic development (Table 1).

The RNAi dataset presented here points to cellular pathways beyond spindle assembly in which *C. elegans* kinesin-5 might play a role. Furthermore, the data on reproductive fitness in the wild type strain N2 may serve as a useful reference for other genome-wide RNAi screens in *C. elegans*. Finally, we hope that our adaptation of 96-well liquid format screening^12^ to an automated pipeline with a microscope image-based read-out will encourage others to execute similar large-scale screens using this approach.

## Methods

### Strains and culture

Worms were cultured on nematode growth media (NGM) plates seeded with OP50 bacteria. Strains used in this study include: N2 wild type Bristol variant, SV1005 *bmk-1(ok391)* V backcrossed 8 times with N2, SV1069 *myo-2::GFP* backcrossed 4 times with N2 and SV1070 *myo-2::GFP*; *bmk-1(ok391)* V from crossing SV1005 with SV1069. Worm strains were grown at 16 or 20 °C. Newly generated strains will be donated and available through the Caenorhabditis Genetics Center (CGC).

### High throughput RNA interference screening

#### Methodology overview

To perform the genome-wide RNA interference (RNAi) screen we devised an automated screening platform, based on a previously developed protocol for *C. elegans* screening in liquid culture using 96-well plates^12^. We used synchronized first larval stage (L1) animals, which we fed with bacteria from the *C. elegans* RNAi feeding library constructed by the Ahringer group^11^. We compared the viability of progeny from control and *bmk-1(ok391)* parents following feeding with bacteria expressing gene-specific double-stranded RNA (dsRNA). For each 96-well library plate with bacteria, we assayed two technical replicates per strain as well as negative and positive controls, positioned in column 1 (wells A1 to H1). The negative controls were empty feeding vector (L4440, a gift from Andrew Fire (Addgene plasmid # 1654)) and *hil-5* dsRNA that exhibit no lethality*. plk-1* dsRNA, which causes 100% embryonic lethality, was used as a control for RNAi, *and dli-1* dsRNA was used as a control for enhanced lethality specifically in the *bmk-1(ok391)* background (hereafter referred to as positive control). Worm strains SV1069 and SV1070 express green fluorescent protein (GFP) in the pharynx from the myo-2 promoter, which allowed image acquisition and worm counting with an automated fluorescence microscope (Thermo ArrayScan VTi) (Fig. 1a). All liquid dispensing steps were carried out with the Sciclone ALH 3000 liquid handling robot (Caliper Lifesciences). L1 larvae were dispensed into each well of 96-well plates, using a Multidrop^TM^ Combi Reagent Dispenser (Thermo Scientific). For the secondary screen, bacterial clones that enhanced lethality above a certain threshold (see below) were re-assayed in three biological replicates using the same experimental procedure of the primary screen, (Fig. 1b).

#### Screening reagents

- Ahringer RNAi feeding library with 16757 bacterial clones^11^ (Source BioScience, Nottingham, GB)
- LB (Luria-Bertani) media plus 100 μg ml^−1^ Ampicillin and 12,5 μg ml^−1^ Tetracyclin hydrocloride
- NGM agar plates seeded with *Escherichia coli* OP50 (available from the *C. elegans* Genetics center) for growing worms
- IPTG 4 mM to induce *lac* operon controlled gene expression in bacteria
- M9 buffer
- Complete S medium plus 10 ml l^−1^ Penicillin-Streptomycin and 50 mg l^−1^ Nystatin
- Bleach solution (10 M NaOH, 4% sodium hypochlorite)
- Tween 20 (P2287, Sigma-Aldrich)
- Tricaine (E10521, Sigma-Aldrich)
- Tetramisole hydrochloride (T1512, Sigma-Aldrich)

### Day 1: RNAi library replication

The 96-well RNAi library plates with bacterial glycerol stocks and the microtiter plates with controls were thawed at room temperature for 10 min prior to use. A 96-pin replicator (BOEKEL), with the first column of pins removed, was used to inoculate 100 μl of LB (supplemented with Ampicillin and Tetracyclin hydrocloride) in each well of a new 96-well plate with bacteria. Positive and negative controls were then added to the first column in each 96-well plate. The first column of every library plate was replicated into a separate 96-well plate for screening. Bacteria were grown overnight (ON) at 37 °C.

### Day 2: Preparation of bacteria and worms

5 μl of the bacterial ON cultures were used to inoculate 400 μl of LB (supplemented with Ampicillin and Tetracyclin hydrocloride) in a 2-ml 96-well plate, followed by ON incubation in a shaking incubator at 220 r.p.m. at 37 °C. Gravid adult worms were washed off NGM agar plates in M9 buffer, pelleted by centrifugation for 1 min at 1,350 r.p.m. and subjected to bleach solution for 5–10 min with occasional vortexing until only embryos remained. Embryos were pelleted by centrifugation for 1 min at 1,350 r.p.m. and resuspended in M9 buffer three times to remove bleach. Embryos were allowed to hatch ON in M9 buffer in a shaking incubator at 200 r.p.m. at 20 °C.

### Day 3: RNAi feeding

L1 worms were resuspended at a concentration of approximately seven larvae per 10 μl of M9 buffer. Tween 20 was added to the worms to a final concentration of 0,01% to avoid worms sticking to the plastic tubing during dispensing. IPTG was added to each well of the bacterial cultures to a final concentration of 4 mM to induce dsRNA transcription during 1 h incubation at 37 °C. Bacteria was then pelleted by spinning at 4,000 r.p.m. for 5 min and subsequently resuspended in 400 μl of Complete S medium plus 4 mM IPTG. 40 μl of each bacterial culture were transferred to flat-bottomed 96-well plates followed by 10 μl of M9 buffer containing the L1 worms. The plates were incubated at 20 °C for 4 days with shaking at 200 r.p.m., allowing sufficient time for the L1 worms to grow to adults and lay eggs, and for the eggs to hatch and develop into larvae.

### Day 7: Phenotype scoring

Prior to image acquisition, 70 μl of worm paralyzing solution (50 μl of complete S medium and 20 μl of 0,7% Tricaine+0,07% Tetramisole) was added to all wells. We imaged the entire well (25 fields per well with a 20x objective) and counted the number of adult worms (parents, bigger pharynxes) and the number of larvae (progeny, smaller pharynxes) using the Cellomics® Target Activation BioApplication (Fig. 1a, magnification).

### Data collection and analysis

#### Quantification of number of adults

**Figure 2:**
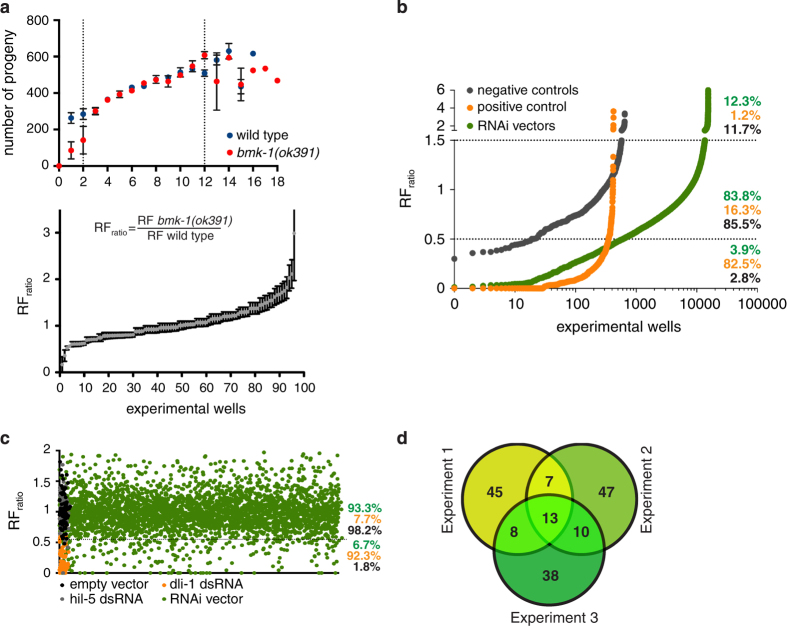
Genome-wide RNAi screen results. (**a, top**) Determination of the ideal number of parents per well. Between 2 and 12 the number of obtained progeny is linear. Wild type strain is *myo-2::GFP*. *bmk-1(ok391); myo-2::GFP* is the mutant strain. (**a, bottom**) RF_ratio_ calculation and distribution of worms treated with an empty RNAi vector. Error bars represent SEM of three biological replicates, each containing two technical replicates. (**b**) Primary screen results. RF_ratio_ distribution of screen controls and library RNAi vectors. Dashed lines represent the thresholds for the definition of ‘enhancers’ (<0.5) and ‘suppressors’ (>1.5). Percentages represent the total number of negative (grey) and positive (orange) controls within each cut-off range. Total number of library RNAi vectors in each category is also represented by percentages (green). (**c**) Secondary screen results. RF_ratio_ distribution of each control or library RNAi vector across three biological replicates. Dashed line indicates the threshold value below which we considered the RNAi to ‘enhance’ *bmk-1(ok391)*. Percentages represent the total number of negative controls (empty vector, *hil-5* dsRNA, in dark grey), positive controls (orange) and library RNAi vectors (green) in each category. (**d**) Venn diagram showing the common and unique hits found within the biological replicates. 38 targeting sequences classify as ‘enhancers’ in at least two out of the three replicates.

Automated image analysis was performed and the number of parent worms, which correspond to the number of L1s dispensed on day 3, was determined. Only wells with 2–12 parent worms were considered for further analysis, as the number of obtained progeny within this range is linear (Fig. 2a, top graph).

#### Determination of reproductive fitness ratio

Reproductive Fitness (RF) is defined as the number of progeny per parent worm treated with dsRNA. It was obtained by dividing the total number of viable progeny by the number of parents for each experimental well. The Reproductive Fitness ratio (RF_ratio_) was obtained dividing the RF of the control strain (SV1070) by the RF of the *bmk-1(ok391)* strain (SV1069) (Fig. 2a, bottom graph).

#### Data import, management, quality assessment and replicate summarization

Raw data, i.e., the RF for each targeted gene in the two strains, was analyzed using the software package cellHTS2 (ref. 13) implemented in Bioconductor/R. Normalization to correct for plate effects was performed by dividing each measurement by the median value across the ‘sample’ wells in each plate.

#### Hits selection. Primary screen

Upon determination of the RF_ratios_, we continued with genes whose depletion led to minimally a 50% increased lethality in SV1070 when compared to SV1069 (RF_ratio_≤0.5) (Fig. 2b). Upon visual inspection of the data, we also included genes whose depletion led to a significant (RF_ratio_≥1.5) reduction in lethality in SV1070 when compared to SV1069. Targeted genes resulting in more than 90% lethality in the control strain were excluded, since the RF_ratio_ would be unreliable. In total 1,057 bacterial clones were selected for the secondary screen (575 enhancers and 482 suppressors) (Data Record 1).

#### Hits selection. Secondary screen

Using the RF_ratio_ of the positive and negative controls, we determined the percentage of expected false positives and false negatives for cut-offs ranging from 40–70%. We decided to continue with bacterial clones showing a minimum increase in lethality of 45% in SV1070 when compared with SV1069 (RF_ratio_<0.55), since this predicts a similar percentage of false positive and false negative results across three biological replicates. 168 bacterial clones classified as hits under this criterion (Fig. 2c) (Data Record 2). Next, we built a Venn diagram with the three sets of hits from the three independent experiments and accepted as final hits genes that classified as enhancers in at least two out of the three independent experiments (Fig. 2d). All targeting vectors from the final hit list were sequenced in order to confirm the identity of the targeted gene (Table 1).

## Data Records

The raw and analyzed data produced by this screen are formatted as two downloadable spreadsheets from FigShare (Data Citation 1).

### Data record 1—primary screen

Primary dsRNA screen data are contained in tabs termed Controls (negative and positive controls), Samples (RNAi vectors) and ND Samples (targeting sequences for which an RF_ratio_ could not be determined for technical reasons). The tab termed ‘Selection 2nd Screen’ contains all the targeting sequences taken to the secondary screen and respective location coordinates in the arrayed hit plates (1–13).

Information regarding the column headings:

#### Plate

Ahringer library plate number.

#### Well

Well coordinates.

#### Description

Content description. ‘sample’, RNAi vector. ‘neg’, negative control. ‘pos’, positive control. ‘other’, embryonic lethality control.

#### GeneID

Gene sequence identification number/name.

#### Raw_r1/r2_

Raw data value for technical replicate 1 or 2 for each assayed worm strain.

#### RF (AVG replicates)

Average (arithmetic mean) Reproductive Fitness of replicates. Sometimes the RF of one of the two replicate could not be determined for technical reasons. In these cases the RF value corresponds to the RF of the single replicate that worked.

#### RFratio

Obtained dividing the RF (AVG replicates) of SV1070 by the RF (AVG replicates) of SV1069.

#### Classification

Controls or Samples are classified as ‘enhancer’ if RF_ratio_ is ≤0.5, ‘suppressor’ if RF_ratio_ is ≥1.5, otherwise ‘no effect’.

### Data record 2—secondary screen

Secondary dsRNA screen data are included under different tabs of the spreadsheet. Content description and column details are given below:

#### ‘Total data’

Contains the raw values and calculated RF_ratios_ for every experimental well of the three independent experiments. Column headings are the same as in Data Record 1.

#### ‘Controls’

The RF_ratio_ for the negative and positive controls from the previous sheet was calculated for each experiment. Next, we classified each result as ‘enhancer’, ‘no effect’ or ‘suppressor’ applying different cut-offs (40%, 45%, 50%, 55%, 60%, 65%, 70%) to the RF_ratios_. We then determined the predicted percentage of false positive and false negative results for each cut-off. To generate the final hit list, we used a 45% cut-off, which predicted a similar percentage of false positive and false negative results across the three independent experiments.

#### ‘Samples’ (45%)

RF_ratio_ for each RNAi vector was extracted from the ‘Total data’ sheet and classified as ‘enhancer’, ‘no effect’ or ‘suppressor’ according to a 45% cut-off. Cells in red correspond to targeting sequences classifying as ‘enhancers/suppressors’ in the three independent experiments. Cells in orange correspond to targeting sequences classifying as ‘enhancers/suppressors’ in two out of the three independent experiments. ‘suppressors’ were not taken into consideration for further analysis, since they were not the aim of this screen. Nevertheless, they might represent a suppression of a RNAi phenotype by the *bmk-1* loss of function.

## Technical Validation

### Technical and biological replicates

It was not possible to do the primary screen in multiple biological replicates due to the high cost and logistic challenges. Therefore, we used two technical replicates. For the secondary screen, three biological replicates were used, each containing two technical replicates. We used the same rational as in the primary screen, and in the end selected hits based on reproducibility.

### Controls

Negative controls were used to define the threshold for hit selection. The positive control was found in a pilot screen.

### Validation of the genetic interactions

**Figure 3:**
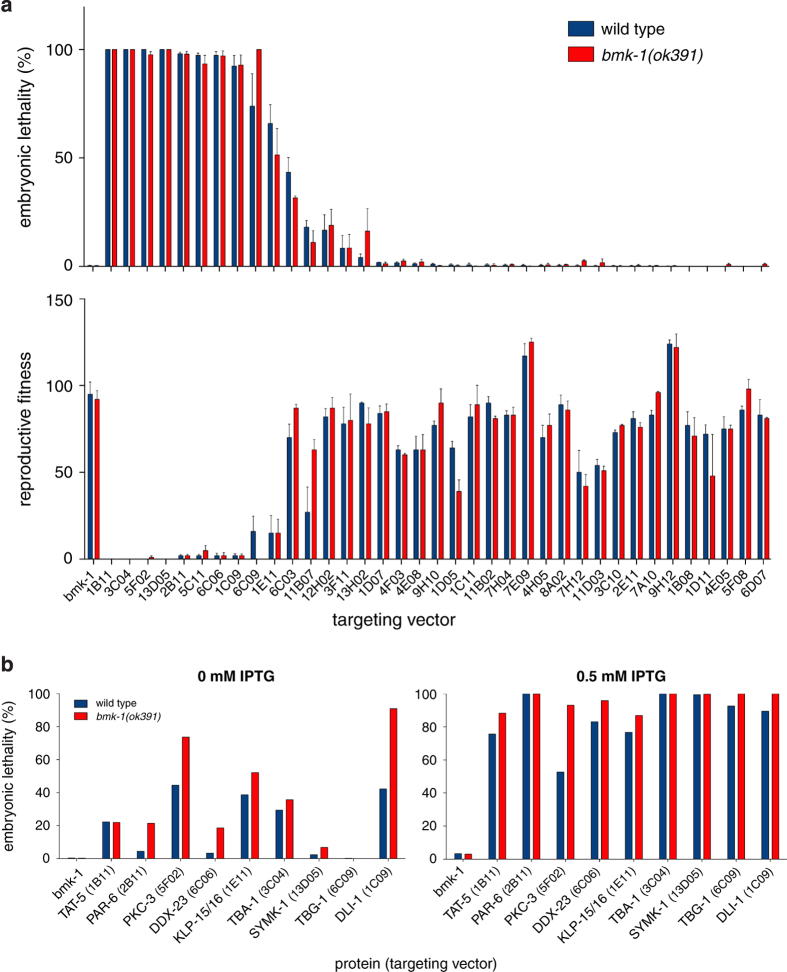
RNAi screen validation. (**a**) Embryonic lethality assay with the screen's ‘enhancers’. N2 (blue), wild type strain. SV1005 (red), *bmk-1(ok391)* deletion strain. RNAi for *bmk-1* was used as a negative control. Error bars represent SEM of three technical replicates. Top graph shows embryonic lethality and below the corresponding reproductive fitness (expressed in number of live progeny per parent). (**b**) Embryonic lethality assay upon weak (0 mM IPTG) or strong (0.5 mM IPTG) depletion of screen ‘enhancers’. Bars represent averages of two technical replicates.

In order to confirm the lethality enhancement when the screen hits (‘enhancers’) are depleted in the *bmk-1(ok391)* background, we performed a classical embryonic lethality assay^14^. Briefly, this assay consisted of feeding wild type (N2) and *bmk-1(ok391)* (SV1005) L4 worms with HT115 bacteria carrying the RNAi vector and inducing the expression of dsRNA by IPTG addition (0.5 mM) on agar plates. These worm strains share the same genetic background as the strains used in the RNAi screen, with the exception that they do not express any fluorescent marker. 24 h after we started feeding the worms with dsRNA at 20 °C, we transferred them to new RNAi plates and allowed egg laying for another 24 h, after which the adult worms were removed. 24 h later embryonic lethality was accessed (Fig. 3a). Several gene knockdowns led to 100% embryonic lethality (e.g., *dli-1, par-6, tbg-1*) already in the wild type strain. This suggested that the knockdown of these proteins on agar plates was more penetrant than in the liquid format. Because this precluded us from detecting enhancement of lethality in the *bmk-1(ok391)* background, we repeated the embryonic lethality assay on agar plates in two conditions: weak RNAi (0 mM IPTG, resulting in partial depletion due to basal transcription of dsRNA in the absence of induction) and strong RNAi (0.5 mM IPTG, corresponding to maximal induction of dsRNA production). We were able to confirm an increase in embryonic lethality when the selected proteins were partially depleted (0 mM IPTG) in the *bmk-1(ok391)* background (Fig. 3b).

Interestingly, 50% of the hits did not show any embryonic lethality when dsRNA was delivered by feeding on plates (Fig. 3a). We reasoned that they were selected as hits in the screen because the depletions caused a significant delay in larval development in the *bmk-1(ok391)* background relative to controls. As the automated analysis counted the number of progeny based on the size of their pharynxes, larvae with small pharynxes would remain undetected, lowering the reproductive fitness ratio. To test this hypothesis, we repeated the RNAi-mediated depletion of these proteins by feeding on plates and classified the phenotype of larval progeny 3 days after removal of the parent. For 14 out of 38 bacterial clones, we observed enhanced growth defects in the *bmk-1(ok391)* background (Table 1). We conclude that the genetic interactions uncovered in this screen include genes with roles in post-embryonic development.

## Usage Notes

The RNAi screen data (Data Records 1–2) are provided for users to be able to apply their own normalisation strategies and thresholds for identifying differences between the two strains. This study focused on genes that when depleted enhanced the lethality in the worm kinesin-5 mutant *bmk-1(ok391)* when compared with the control strain. However, some genes showed decreased lethality when depleted in the *bmk-1(ok391)* background. These genes are also potential interactors of kinesin-5. Furthermore, several dsRNAs led to a significant developmental delay specifically in the *bmk-1(ok391)* background, which may indicate a function of kinesin-5 during development. The results obtained in this screen can be compared with data from previous RNAi screens in *Caenorhabditis elegans* as reported in RNAiDB (ref. 15).

## Additional information

**How to cite this article:** Maia, A.F. *et al.* Genome-wide RNAi screen for synthetic lethal interactions with the *C. elegans* kinesin-5 homolog BMK-1. *Sci. Data* 2:150020 doi: 10.1038/sdata.2015.20 (2015).

## Supplementary Material



## Figures and Tables

**Table 1:** Final hit list

**hit plate**	**well**	**Cosmid(s)**	**Primary targets**	**Secondary targets**	**Gene(s) name(s)**	**N2 (wild type)**		**SV1005 (kinesin-5 mutant)**	
						**Embryonic**	**Post-embryonic**	**Embryonic**	**Post-embryonic**
1	B08		R06C7.10		myo-1		Gro		Gro
1	B11	F36H2	F36H2.1		tat-5	Emb		Emb	
1	C09	C39E9+B0564	C39E9.14		dli-1	Emb	Adl+Ste+Bag	Emb	Ste+Pvl
1	C11	F46A9+F30A10	F46A9.4	F46A9.5	skr-2+skr-1		Gro		Gro
1	D05	W09C3+T03F1	W09C3.6	T03F1.5	gsp-3+gsp-4		Gro		Gro
1	D07	W01A8	W01A8.4		nuo-6		Lva		Lva
1	D11	C17E4	C17E4.5		pabp-2		Gro+Lva		Gro+Lva
1	E11	C41G7+M01E11	C41G7.2	M01E11.6	klp-16+klp-15	Emb	Gro	Emb	Gro
2	B11	T26E3	T26E3.3		par-6	Emb	Ste	Emb	Ste
2	E11	T08B2	T08B2.8	T08B2.7	mrpl-23+ech-1.2		Gro		Gro
3	C04	F26E4	F26E4.8		tba-1	Emb		Emb	
3	C10	Y71F9AM	Y71F9AM.4	Y71F9AM.7	cogc-3+Y71F9AM.7		Ste+Sck		Ste+Sck
3	F11	F52C6+M7	M7.1	F52C6.12	ubc-2(let-70)+F52C6.12	Emb	no defect	Emb	no defect
4	E05	F37C12	F37C12.3		F37C12.3		Gro		Gro
4	E08	B0285	B0285.1		cdtl-7		Lva		Lva
4	F03	T12G3	T12G3.5		mrpl-51		Gro+Lva		Gro+Lva
4	H05	B0280	B0280.9		B0280.9		Gro+Ste+Sck		Gro+Ste+Sck
5	C11	W09H1+several other			several his- genes	Emb		Emb	
5	F02	F09E5	F09E5.1		pkc-3	Emb		Emb	
5	F08	Y49E10+Y51H7C+ Y9D1A+Y46H3C+etc					Gro		Gro
6	C03	several	F54D5.11	T01G5.8		Emb	Gro+Rup	Emb	Gro+Rup+Pat
6	C06	F01F1	F01F1.7		ddx-23	Emb	Lva	Emb	Lva
6	C09	F58A4	F58A4.8		tbg-1	Emb	Ste+Pvl	Emb	
6	D07	Y43C5A	Y43C5A.6		rad-51		no defect		no defect
7	A10	H06I04	H06I04.3		H06I04.3		Gro		Gro+Ste
7	E09	M04D8	M04D8.6		xbx-3				no defect
7	H04	F42A8+C06C3	C06C3.1		mel-11		Adl+Ste+Bag		Ste+Sck
7	H12	E04A4	E04A4.7		cyc-2.1		Gro		Lva
8	A02	K08F11	K08F11.4		yars-1		Gro		Gro+Lva
9	H10	YAC Y38F2AL	Y38F2AL.4		vha-3		Lva		>Gro
9	H12	Y6E2A	Y6E2A.9		sfxn-1.3		Gro+Lva		no defect
11	B02	Y82E9BR	Y82E9B5.3		Y82E9BR.3+Y82E9BR.27+Y82E9BR.30		Gro		>Gro
11	B07	F26H11	F26H11.1		kbp-3 (knl)	Emb	Ste	Emb	Ste+Adl+Gro+Pvl
11	D03	F11A3+C50F4	F11A3.2		F11A3.2		Gro		Ste+Lva
12	H02	T27B1	T27B1.2		ztf-19/pat-9	Emb		Emb	Pat+Adl+Rup+Dpy
12	F04	T27B1	T27B1.2		ztf-19/pat-9	Emb	Ste+Sck	Emb	
13	D05	F25G6	F25G6.2		symk-1	Emb	no defect	Emb	Gro+Sck+Adl
13	H02	F49C12	F49C12.13		vha-17		Gro		
Table summarizing the genes that genetically interact with the worm kinesin-5 and the observed developmental defects upon dsRNA treatment. Description of the abbreviations can be found at www.RNAi.org^15^.									
